# Material Performance and Animal Clinical Studies on Performance-Optimized Hwangtoh Mixed Mortar and Concrete to Evaluate Their Mechanical Properties and Health Benefits

**DOI:** 10.3390/ma8095306

**Published:** 2015-09-17

**Authors:** Bon-Min Koo, Jang-Ho Jay Kim, Tae-Kyun Kim, Byung-Yun Kim

**Affiliations:** 1 Concrete Structural Engineering Laboratory, School of Civil and Environmental Engineering, Yonsei University, Seoul 120-794, Korea; E-Mails: kbm255@naver.com (B.-M.K.); ssida24@naver.com (T.-K.K.); 2 Department of Architectural Engineering, Catholic Kwandong University, Gangneung 210-701, Korea; E-Mail: kby@cku.ac.kr

**Keywords:** Hwangtoh, health benefits, Institute for Cancer Research (ICR), material properties, mix proportion design

## Abstract

In this study, the amount of cement used in a concrete mix is minimized to reduce the toxic effects on users by adjusting the concrete mixture contents. The reduction of cement is achieved by using various admixtures (ground granulated blast-furnace slag, flyash, ordinary Portland cement, and activated Hwangtoh powder). To apply the mix to construction, material property tests such as compressive strength, slump, and pH are performed. Preliminary experimental results showed that the Hwangtoh concrete could be used as a healthy construction material. Also, the health issues and effects of Hwangtoh mortar are quantitatively evaluated through an animal clinical test. Mice are placed in Hwangtoh mortar and cement mortar cages to record their activity. For the test, five cages are made with Hwangtoh and ordinary Portland cement mortar floors, using Hwangtoh powder replacement ratios of 20%, 40%, 60%, and 80% of the normal cement mortar mixing ratio, and two cages are made with Hwangtoh mortar living quarters. The activity parameter measurements included weight, food intake, water intake, residential space selection, breeding activity, and aggression. The study results can be used to evaluate the benefits of using Hwangtoh as a cement replacing admixture for lifestyle, health and sustainability.

## 1. Introduction

A recent study by the city of Seoul, South Korea, stated that foreign and domestic ordinary Portland cement (OPC) products emitted 4.14 ppm and 13 ppm, respectively, of poisonous hexavalent chromium Cr(VI). Thus, domestic OPC is 3.25 times more hazardous than foreign OPC [1]. Since 2009 in Korea, the maximum domestic and international allowable amount of Cr(VI) in OPC cement is 20 mg/kg and 2 mg/kg, respectively. It has been reported that Hwangtoh (red clay) does not contain Cr(VI) substance [2]. Such concerns have led to a rise in the use of activated Hwangtoh as an eco-friendly construction material. As a result, much research has been conducted on Hwangtoh-mixed construction materials. However, most of it has focused on differences in the material and structural performance capacities of OPC and Hwangtoh; unfortunately, no study has yet quantified the health benefits of Hwangtoh-mixed construction materials. Published reports have considered the activation method of kaolin and activated kaolin-mixed mortar concrete as well as the development of Hwangtoh admixtures for cement mortars [3,4,5,6]. A Hwangtoh cement mortar study analyzed the effect of various firing temperatures on the compressive strength of Hwangtoh mortars and Hwangtoh concrete, suggesting an optimum firing temperature range of 500 °C–1000 °C. Moreover, the same study reported that Hwangtoh serves as an appropriate cementitious material, because its performance equals or exceeds that of other materials, such as blast furnace slag micropowders and flyash [7,8,9]. Based on mineralogical categorization, Hwangtoh is defined as a Halloysite in the kaolin or China clay group, composed mainly of SiO_2_, Al_2_O_3_, and Fe_2_O_3_ [3]. Hwangtoh behaves similarly to concrete admixtures and has the pozzolanic reaction when mixed with cement and water [10,11]. To increase the usability of Hwangtoh as a construction material, activated Hwangtoh is produced by burning natural Hwangtoh at temperatures of 550 °C–1000 °C and quickly cooling it before grinding it into powder form [12]. The published studies report that partial replacement of cement with activated Hwangtoh produces Hwangtoh mortar or Hwangtoh concrete with sufficient strength for possible use as a construction material [13]. A study evaluated the workability, compressive strength, inelastic deformation, and hydration heat of concrete mixed with Hwangtoh or blast furnace slag micropowders by analyzing stress–strain relations, flexural behavior, dry shrinkage, and the creep characteristics of reinforced concrete beams mixed with Hwangtoh or blast furnace slag [12,14]. According to that study, the long-term behaviors of concrete mixed with Hwangtoh or blast furnace slag, such as creep and shrinkage, vary depending on the choice of cementitious material. The dry shrinkage increases when Hwangtoh alone is used, but decreases when both Hwangtoh and blast furnace slag are used. Furthermore, an examination of Hwangtoh mortars without cement revealed that Hwangtoh increased drying shrinkage by 20% [15]. A study of the cytotoxicity of recycled OPC mortar powder reported that the tested powders exhibit a strong and concentration-dependent cytotoxicity of 75.5% [16]. A study assessed the effect of grouting materials on pollution by examining the fish toxicity of those materials [17]. Our review of the literature showed that most studies have focused on structure and durability issues that arise from adding a Hwangtoh admixture, without consideration for any potential health benefits from its use. Meanwhile, in Japan, vigorous research has been conducted on the harmful properties of construction materials such as concrete, metal, and timber. A study found that the survival rates of experimental subjects were 7%, 41%, and 81% for concrete, metal, and timber, respectively, showing that concrete had the lowest survival rate [18]. Thus, in the present study, a preliminary experiment is conducted to determine the appropriate ratio of Hwangtoh to cement and identified the properties of Hwangtoh mixed concrete. Then, a human health benefit assessment is conducted using ICR mice in cages built with either Hwangtoh mixed mortar or cement mortar [19]. We made one cage with a cement mortar floor and four cages with Hwangtoh mixed mortar floors with varying ratios of Hwangtoh to cement (20%, 40%, 60%, and 80%) and two cages with cement mortar and Hwangtoh cement mortar walls and floor to assess the experimental subjects’ choice of living environment. In addition, the subjects’ weight change, food intake, water intake, residential environment preference, fertility rate, and aggression are measured to evaluate the human health benefits of the different materials. The ultimate objective of this study was to develop environmentally friendly construction materials.

## 2. Material Properties of Activated Hwangtoh Mixed Concrete

### 2.1. Reaction Mechanism

Hwangtoh in its natural state has a very low reactivity. However, when heated at high temperatures and cooled rapidly, it is activated by the preserved crystallization energy and holds latent hydraulic properties that harden under certain conditions. The chemical composition of the Hwangtoh we used is shown in Table 1. When Hwangtoh is activated by heating at 550 °C–950 °C, it transitions into an excited state of high pozzolanic reactivity, but it does not have a hydration reactivity when in contact with water. However, Hwangtoh does initiate hydration under alkaline conditions. Concrete is a natural alkaline material; thus mixing activated Hwangtoh in concrete will induce pozzolanic reactions. The activated Hwangtoh reacts with calcium hydroxide (Ca(OH)_2_), the hydration product of cement, and acquires latent hydraulic properties that in turn enhance the strength and water resistance of the concrete. Furthermore, mixing activated Hwangtoh into cement blocks reduces the size of internal voids by filling the spaces between the binder particles, which increases the contact area between the binder and the aggregate, reducing overall concrete bleeding and segregation. At room temperature, silicon dioxide (SiO_2_), a component of activated Hwangtoh, binds with Ca(OH)_2_ and produces a stable pozzolanic product that holds the same properties as other pozzolanic materials, such as blast furnace slag and flyash. Activated Hwangtoh is a material that under goes pozzolanic reaction. It has been reported that Hwangtoh in natural and activated state does not and does have pozzolanic activity, respectively [20]. However, it has not been completely proven that activated Hwangtoh has hydration capacity. From a published report on the topic, the measured hydration temperature of concrete mixed with activated Hwangtoh powder and OPC (41.0 °C) was slightly lower than that of 100% OPC concrete (56.4 °C), indicating that less hydration reaction has taken place by replacing a portion of OPC with activated Hwangtoh powder [21,22,23]. Therefore, a further study on this topic must be performed.

**Table 1:** Chemical composition of activated Hwangtoh and OPC.

Component	Unit Mass Percentage (%)	
	Hwangtoh	Cement
SiO_2_	57.7	22.0
Al_2_O_3_	29.5	5.80
Fe_2_O_3_	6.32	3.30
K_2_O	3.54	–
TiO_2_	1.12	–
MgO	1.06	1.20
F	0.22	–
CaO	0.126	64.04
Na_2_O	0.114	–
P_2_O_5_	0.0620	–
MnO	0.0518	–
SO_3_	0.0434	–

Depending on the product formed by the reaction between activated Hwangtoh and Ca(OH)_2_, the reaction can be classified as pozzolanic or stratlingite. A pozzolanic reaction between SiO_2_ (Hwangtoh) and Ca(OH)_2_ (cement) produces the pozzolanic product afwillite. The equation for the reaction is shown in Equation (1):

3Ca(OH)_2_ + 2SiO_2_ → 3CaO·2SiO_2_·3H_2_O (1)

SiO_2_ and aluminum oxide (Al_2_O_3_) from Hwangtoh and Ca(OH)_2_ from cement can also react to form stratlingite, which makes the activated Hwangtoh into a dense mortar. The unstable Ca(OH)_2_ becomes stable after undergoing the stratlingite reaction, which leads to increased long-term strength, durability, and crack resistance in the concrete. The activated Hwangtoh we used was a product of the Gochang region of Korea composed of natural-state Hwangtoh sintered at 850 °C. The specific gravity and fineness of the activated Hwangtoh were 2.7 cm^2^/g and 3200 cm^2^/g, respectively. The chemical composition of activated Hwangtoh and OPC are shown in Table 1.

### 2.2. Calculating the Replacement Ratio of Activated Hwangtoh

**Figure 1:**
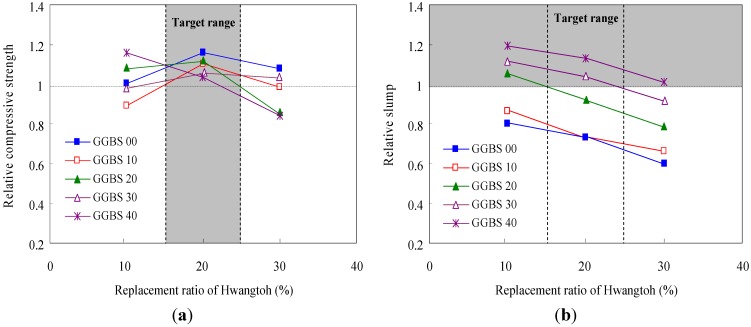
Relative compressive strength and slump test results on concrete samples. (**a**) Actual strength/required strength ratio and (**b**) Actual slump/required slump ratio.

Activated Hwangtoh absorbs more water than ordinary concrete. Therefore, the appropriate mixture proportion based on workability needed to be selected. The cement used in this study had a specific gravity of 3.16. As required by KS L ISO 679 [24], a standard aggregate produced for concrete and mortar with specific gravity of 2.6 and 2.7, respectively, was used for the fine aggregate. The coarse aggregate was a crushed stone aggregate with a maximum diameter of 25 mm and saturated surface-dry specific gravity of 2.65. In addition, to determine the appropriate mixture ratio of activated Hwangtoh, the compressive strength was measured by setting the water-to-binder (W/B) ratio at 43% and increasing the proportion of blast furnace slag from 0%–40% in 10% increments. As shown in Figure 1a, the proportion of activated Hwangtoh ranged from 15%–25%. Because concrete mixed with activated Hwangtoh has a lower compressive strength than ordinary concrete, a 20% ratio of activated Hwangtoh was chosen to secure maximal compressive strength. Figure 1b displays the changes in slump for the different replacement ratios.

### 2.3. Slump Test

Based on the KS F 2401 [25] and KS F 2402 [26] testing methods, the slump was measured by testing a sample of ordinary concrete and activated Hwangtoh mixed concrete. The ordinary concrete showed a slump of 10 cm–13 cm, whereas the activated Hwangtoh mixed concrete showed a slump of less than 1.0 cm and 3.5 cm for a W/B ratio of 43% and 45%, respectively. The slump was enhanced when the W/B ratio was set to 50%. However, the 28 day compressive strength of the concrete with a 50% W/B ratio and 20% activated Hwangtoh was significantly lower. To generate an appropriate slump, a polycarbonate-based superplasticizer was added at increasing ratios from 1%–2% in 0.5% increments. At 2%, the required slump was obtained, but concrete segregation occurred. The most appropriate proportion of superplasticizer for the required strength and slump was 1.5%. Table 2 shows the changes in slump for varying proportions of superplasticizer in the 20% activated Hwangtoh concrete mixture with a 45% water-to-binder (W/B) ratio.

**Table 2:** Slump test results.

Water-to-binder ratio (%)	Hwangtoh replacement ratio (%)	Plasticizer (%)	Slump (cm)
45	Control	–	13
	20	–	3.5
		1	6
		1.5	9–10
		2	15–18

### 2.4. Compressive Strength

For the preliminary test, concrete with 20% activated Hwangtoh, three W/B ratios (43%, 45%, and 50%), and varying proportions of blast furnace slag and flyash (5%–25%) were used. Table 3, Table 4 and Table 5 show the mixture proportions of Hwangtoh concrete. The air content of the fresh concrete was measured using the pressure method in KS F 2421 [27], which showed that it satisfied the criteria of 4.5%. The slump range and air content was set to 12 ± 2.0 mm and 4.5 ± 0.5%, respectively. G in Table 3 refers to the ground granulated blast furnace slag (GGBS) and F refers to the flyash. Twelve specimens were prepared for each case and wet cured each specimen at 20 °C in accordance with KS F 2403 [28]. Blast furnace slag with a specific gravity and fineness of 2.90 cm^2^/g and a 4306 cm^2^/g, respectively, were used. In Table 3, Hwangtoh, water, cement, fine aggregate (sand), coarse aggregate (gravel), GGBS, flyash, and fine aggregate/total aggregate ratio are abbreviated HT, W, C, S, G, GGBS, F, and S/a, respectively.

**Table 3:** Mix proportion of Hwangtoh concrete (W/B 43%, S/a 40%, HT 20%).

Specimen	Unit (kg/m^3^)						
	W	C	S	G	HT	GGBS	F
Control	176	409	679	1034	–	–	–
G 5F25		204.5			81.8	20.45	102.3
G10F20						40.9	81.8
C15F15						61.35	61.35
G20F10						81.8	40.9
G25F 5						102.3	20.45

**Table 4:** Mix proportion of Hwangtoh concrete (W/B 45%, S/a 40%, HT 20%).

Specimen	Unit (kg/m^3^)						
	W	C	S	G	HT	GGBS	F
Control	176	391	681	1042	–	–	–
G 5F25		195.5			78.2	19.55	97.75
G10F20						39.1	78.2
C15F15						58.65	58.65
G20F10						78.2	39.1
G25F 5						97.75	19.55

**Table 5:** Mix proportion of Hwangtoh concrete (W/B 50%, S/a 40%, HT 20%).

Specimen	Unit (kg/m^3^)						
	W	C	S	G	HT	GGBS	F
Control	176	352	694	1062	–	–	–
G 5F25		176			70.4	17.6	88
G10F20						35.2	70.4
C15F15						52.8	52.8
G20F10						70.4	35.2
G25F 5						88	17.6

Flyash with a specific gravity and fineness of 2.19 cm^2^/g and 3.490 cm^2^/g, respectively, was used to mix the concrete in accordance with KS L 5405 [29]. The flyash was incorporated to improve the workability, strength, and durability of the Hwangtoh mixed concrete by reducing the heat of hydration, increasing the water permeability, and increasing the corrosion and chemical resistance. Although several types of superplasticizers are available, such as naphthalene, melamine, and aminosulfonate, the polycarbonate superplasticizer we used is the most popular option for concrete because of its excellent cement dispersing and slump maintaining characteristics. Moreover, polycarbonate superplasticizer is less vulnerable than other superplasticizers to changes in physical properties over time. Therefore, it was used to increase the workability of Hwangtoh mixed concrete. The particular superplasticizer used in this experiment had a dark brownish color with a specific gravity of 1.080 and a pH of 7.0 at 20 °C.

**Figure 2:**
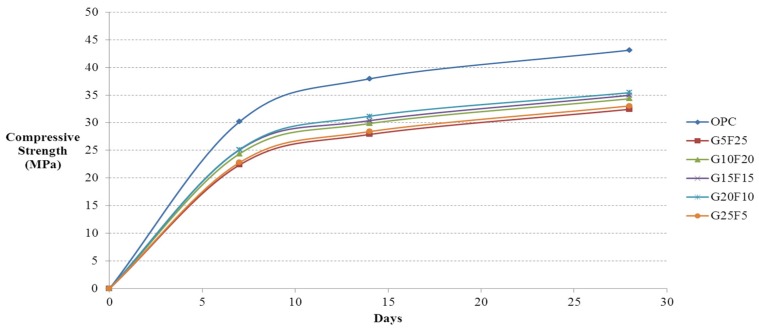
Compressive strength history of concrete with GGBS and flyash (W/B 43%).

**Figure 3:**
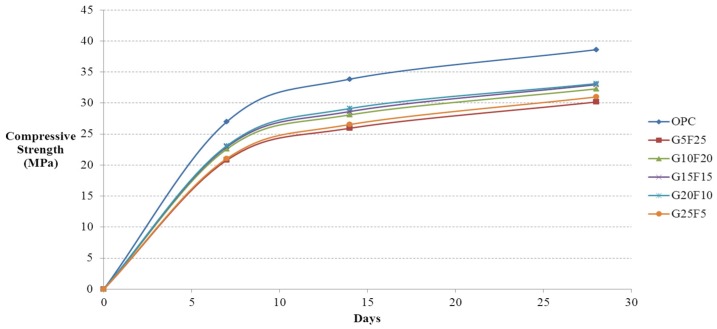
Compressive strength history of concrete with GGBS and flyash (W/B 45%).

**Figure 4:**
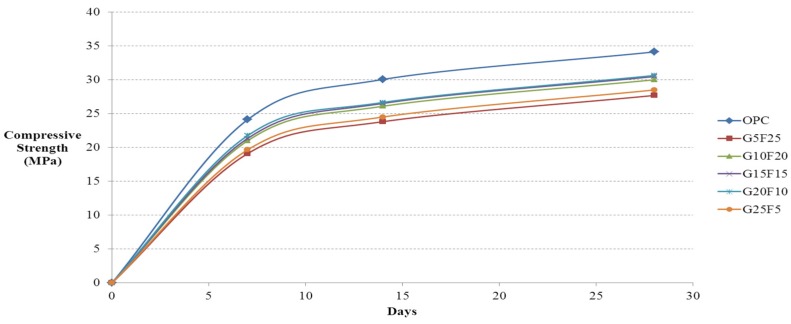
Compressive strength history of concrete with GGBS and flyash (W/B 50%).

**Figure 5:**
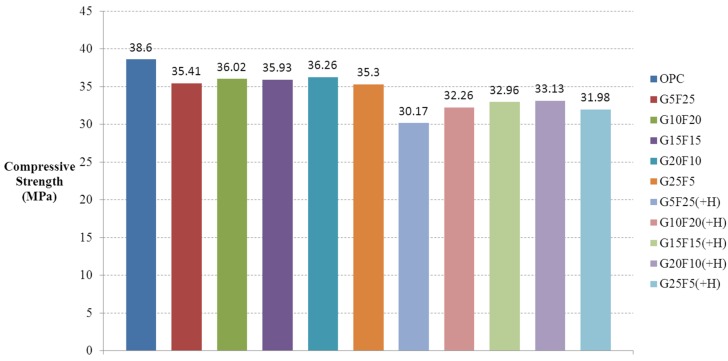
Compressive strength test results (W/B 45%).

After casting 12,100 mm × 200 mm cylindrical specimens using activated Hwangtoh mixed concrete, their compressive strengths on the 7th, 14th, and 28th days from casting were measured in accordance with KS F 2403 [28] and KS F 2405 [30]. The lowest and highest measured values were eliminated and calculated the average compressive strength with the remaining 10 specimens. Figure 2 shows the compressive strength test results for ordinary concrete and concrete mixed with 20% activated Hwangtoh, both with a W/B ratio of 43%. Figure 3 and Figure 4 show the compressive strength test results of the same concrete with a W/B ratio of 45% and 50%, respectively. The compressive strength increased as the W/B ratio decreased. With the same W/B ratio, the differences in the compressive strengths of ordinary concrete and activated Hwangtoh mixed concrete were 7.69 MPa, 5.47 MPa and 3.48 MPa for W/B ratios of 43%, 45% and 50%, respectively. The differences in the compressive strength decreased as the W/B ratio increased. Concrete with 20% blast furnace slag and 10% flyash showed the highest compressive strength, and concrete with 25% blast furnace slag and 5% flyash had the lowest compressive strength. Further follow-up studies are required to better examine the causes of these differences in compressive strength. Figure 5 shows the 28-day compressive strength test results for ordinary concrete and Hwangtoh mixed concrete with a W/B ratio of 45%.

### 2.5. pH

**Figure 6:**
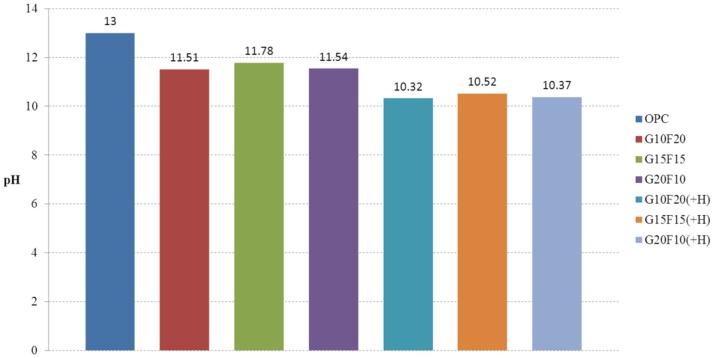
pH test results (W/B 45%).

A pH150-C was used to measure the pH. This equipment can measure a pH range from 0–14. Flat surface electrodes were attached to the surface of the activated Hwangtoh mixed concrete specimens and measurements at three points from each specimen were obtained to calculate the mean. The pH values of the ordinary concrete, the concrete without activated Hwangtoh, and the activated Hwangtoh mixed concrete with W/B ratio of 45% were obtained and are shown as a bar graph in Figure 6. The pH of the ordinary concrete used for construction ranges from 12.5 to 13. The OPC measured in the present study had a pH of 13. The pH of the concrete without activated Hwangtoh ranged from 11.51 to 11.78, depending on the proportions of blast furnace slag and flyash. The pH of the activated Hwangtoh mixed concrete was 10.32–10.50. The Ca(OH)_2_ produced by the hydration reaction with the cement forms a passive film that protects the rebar. However, if it loses alkalinity by binding to CO_2_, it becomes carbonated and forms CaCO_3_ with a pH of 8–9. The pH of the activated Hwangtoh mixed concrete was 1.19–1.28 lower than that of the concrete without activated Hwangtoh; the pH of the ordinary concretes was 1.25–1.54 higher than that of the activated Hwangtoh mixed concretes. These results indicate that users must pay meticulous attention not to expose activated Hwangtoh mixed concrete to substandard conditions. From a published report, poisonous and beneficial effects of OPC and Hwangtoh porous concrete on living insects, respectively, were found to be due to high and low pH property, respectively [31]. Therefore, it is safe to assume that Hwangtoh contains less harmful substances than OPC cement with regards to health issues.

## 3. Health Benefit Assessment of Activated Hwangtoh Mixed Concrete

**Figure 7:**
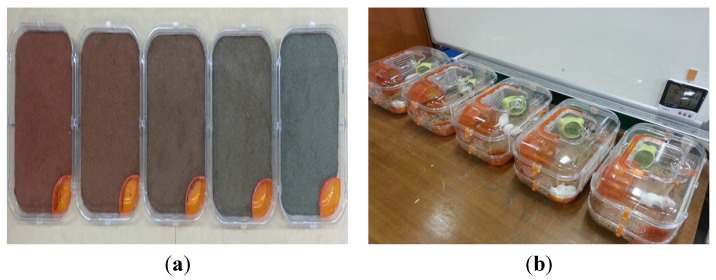
Floor cage photos. (**a**) Casted bottom surfaces and (**b**) Floor cages.

**Figure 8:**
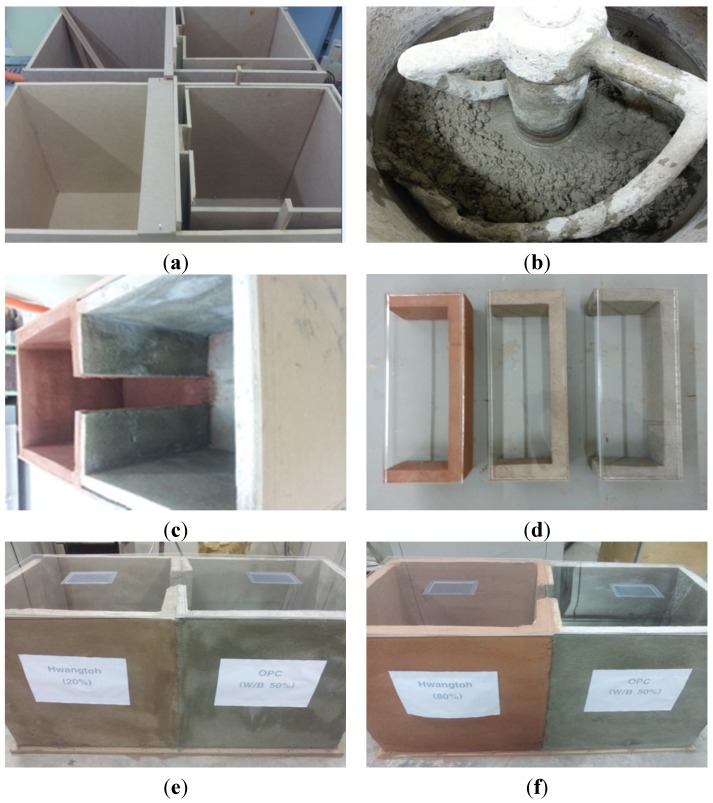
Residence cage photos and specimen types. (**a**) Residence cage forms; (**b**) Concrete mixing; (**c**) Casting and curing; (**d**) Sleeping spaces; (**e**) 20% Hwangtoh and OPC mortar, and (**f**) 80% Hwangtoh and OPC mortar.

The health benefit assessment was performed on three-week-old ICR mice, which are frequently used in animal clinical studies. All experimental procedures complied with the Institutional Animal Care and Use Committee (IACUC) mice standard operating guidelines, the Korean Food and Drug Administration guidelines for animal experimentation, and manuals on experiments involving animals [32]. The cage floors with different ratios of Hwangtoh to cement (20%, 40%, 60%, 80%) with a thickness of 3 cm were prepared, as shown in Figure 7. The titles for the floors made with 20%, 40%, 60%, and 80% Hwangtoh concrete are floor 20, floor 40, floor 60, and floor 80, respectively, and the title of OPC concrete floor is floor OPC. All of the mice were kept at the same temperature and humidity and provided with equal amounts of food and water. To alleviate their stress, exercise equipment was installed in the cage. For the comparison of residential environments, two casts with a length of 122.6 cm, a width of 51 cm, a height of 62 cm, a floor thickness of 5 cm, and a wall thickness of 3 cm were manufactured. Exactly half of each cast was poured with ordinary cement mortar, and the other half was poured with a mixture containing either 20% or 80% activated Hwangtoh, as shown in Figure 8. Therefore, the title of the cast with 20% Hwangtoh concrete and OPC concrete are H20 and OPC20, respectively. Likewise, 80% Hwangtoh and OPC concrete are titled H80 and OPC80, respectively. A passageway connecting the two casts allowed the mice to move freely to either side. The mortar’s mixture ratio was: 0.5 (water):1 (cement):3 (fine aggregate). One pair of male and female ICR mice were placed in each of the five casts for the floor experiment, and five male and five female ICR mice were placed in each of the two casts for the residential selection experiment.

## 4. Temperature and Humidity Changes Depending on Mixture Ratios

**Figure 9:**
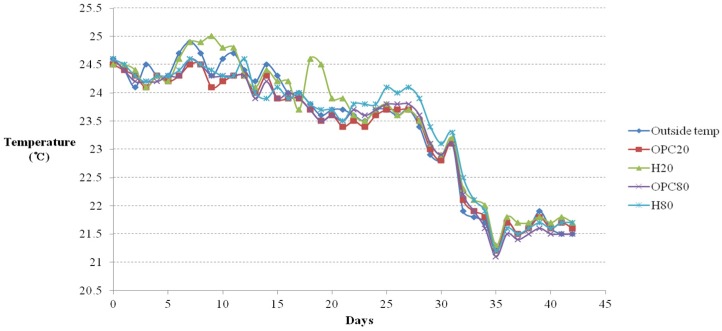
Temperature *versus* time curves.

**Figure 10:**
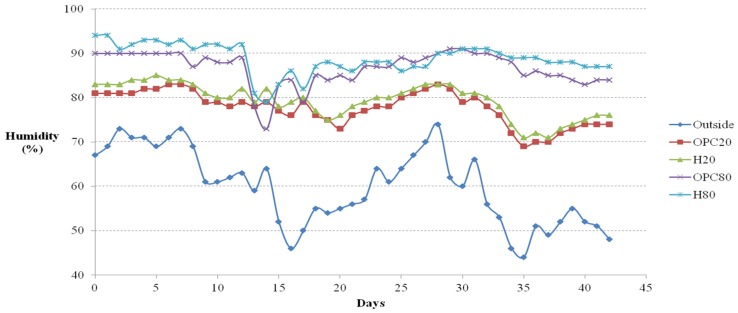
Relative humidity *versus* time curves.

The external and internal temperatures of each cage were measured at the same time daily, as shown in Figure 9 and Figure 10, respectively. From day 21 onwards, the internal temperature surpassed the external temperature because day 21 marked the beginning of winter. Until day 21, the internal temperature was the highest in cast H20; after day 21, the internal temperature was higher in H80. By and large, the temperature was higher in the casts prepared with Hwangtoh concrete than in the OPC concrete casts. The temperature within the cast of OPC20 was higher than in that of OPC80. Under equal external temperatures, the differences in internal temperature and humidity in the activated Hwangtoh mixed environment and the OPC environment ranged from 0.1 °C to 1.5 °C and 1% to 5%, respectively. Although those differences are small, the ICR mice were sensitive to them. Moreover, the humidity of the external environment was lower than that in any internal environment throughout the duration of the study. It was the highest in the internal environment of the H80 cast, followed by the OPC20, H20, and OPC80 casts. The humidity within the OPC20 and H20 casts fell sharply between days 15 and 17, because the acrylic ceilings of the casts were damaged. The standard IACUC test guideline for live animals states that the relative humidity required for ICR mice is 80%. As shown in Figure 10, the H20 and H80 casts satisfied that requirement. However, the external relative humidity was 45%–75%, less than the required 80%. The relative humidity measurements show that the Hwangtoh environment was more effective in maintaining an appropriate relative humidity than an ordinary air environment.

### 4.1. Amount of Food and Water Intake

**Figure 11:**
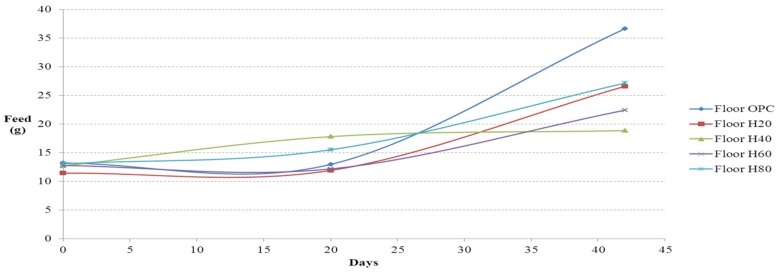
Food intake *versus* time curves (floor cages).

**Figure 12:**
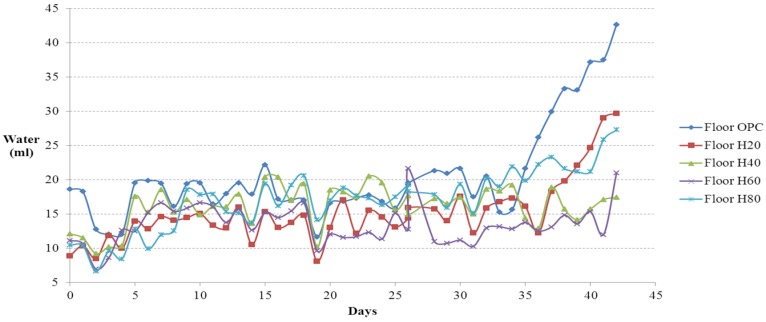
Water intake *versus* time curves (floor cages).

**Figure 13:**
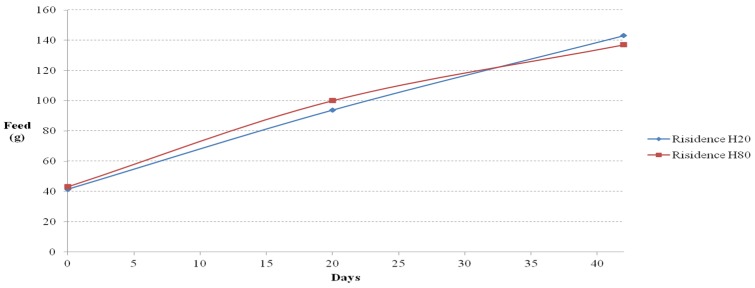
Food intake *versus* time curves (residence cages).

**Figure 14:**
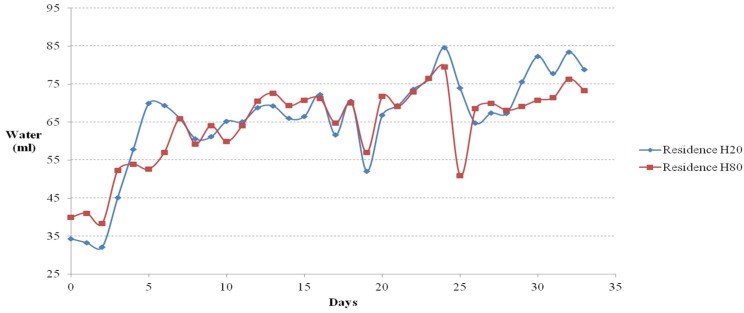
Water intake *versus* time curves (residence cages).

The amounts of food and water intake measured daily from the floor cage test are shown in Figure 11 and Figure 12, respectively. The amounts of food and water intake measured daily from the residential selection casts are shown in Figure 13 and Figure 14, respectively. The amounts were calculated by taking the difference between the initial weights of food and water and the weight of leftover amounts after 24 hours. Small food is difficult to distinguish from mouse feces; therefore, the ICR mice was fed with solid cylindrical food. As shown in Figure 11, from day 1 to 21, the amount of food intake in the floor experiment was the highest on floor 80 (80% of activated Hwangtoh), followed by floor 20, floor OPC, and floor 60. The amount of food intake initially increased moderately and reached a plateau in floor 40, possibly because the ICR mice on the floor 40 cast were infertile. From day 21 onwards, the amount of food intake was the highest on floor OPC, followed by floor 80, floor 20, floor 60, and floor 40. This trend can be attributed to the onset of colder weather on day 21, during which the mice gave birth, thereby increasing the total amount of food intake. Given the design of this study, the cause of the increased food intake in floor OPC cannot be pinpointed to a change in temperature or humidity or the increased desire for reproduction in a substandard environment. A more in-depth anatomical experiment is required to understand this trend. The ICR mice changed their food and water intake before and after parturition; the mice consumed more water and food during pregnancy and less after giving birth. The large food intake of mice on the OPC floor might be attributable to their survival instincts. The relatively lower temperature and humidity of the OPC floor might cause ICR mice to consume more food and water to supplement their energy levels and thus become stronger to overcome their harsh environment.

The ICR mice in the residential selection experiment showed similar upward trends in their amount of food and water intake. Before day 32, the amount of food intake increased in the mice in H80, and after day 32, the amount of food intake increased in the mice in OPC80.

In the flooring experiment, the amount of water intake was the highest in the mice on floor OPC throughout the entire study duration. The mice on floor 40 showed a consistent and repetitive trend. The next highest water intake was by the mice on floor 80, followed by floor 20, floor 40, and floor 60. In the residential selection experiment, the amount of water intake followed a nearly identical upward trend with minor differences in each cast.

### 4.2. Weight Gain

**Figure 15:**
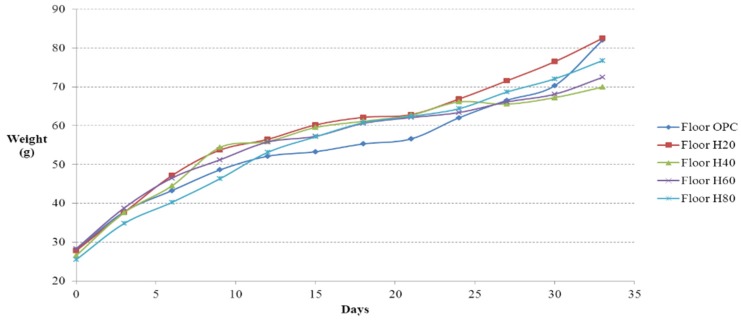
Weight change *versus* time curves (floor cages).

**Figure 16:**
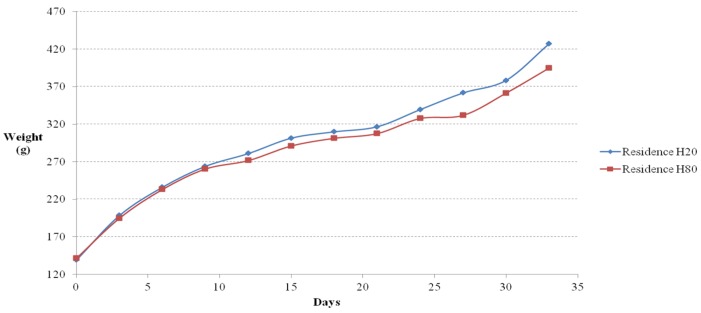
Weight change *versus* time curves (residence cages).

Weight gain at three-day intervals was measured as mandated by the regulations in the IACUC guidelines for animal experimentation. The weight changes measured from the floor cages and residence selection casts are shown in Figure 15 and Figure 16, respectively. In the flooring experiment, the mice on floor 20 showed the highest weight gain; the mice on floor 80 initially showed the lowest weight gain, but they ended up with the greatest weight gain toward the end of the experiment. The weight gain by the mice on floor OPC was moderate in the initial and middle stages of the experiment but reached that of the mice on floor 20 by the end of the experiment. The mice on floor 40 and floor 60 showed identical upward trends in weight gain. Thus, the ICR mice on the concrete floor casts took in large amounts of food and water in the early and middle stages of testing to survive in their new environment. However, as they adjusted to the environment in the later stage of testing, their weight gain increased significantly. However, the ICR mice on the Hwangtoh floor casts took in less food and water than the mice on the concrete floor because of the better environmental conditions provided by the Hwangtoh environment throughout the testing. As shown in Figure 16, the residential selection experiment showed that all of the mice initially had similar weight gains, but the mice in H20 had a higher weight gain than the other mice from the middle to the end of testing.

## 5. Aggression

**Figure 17:**
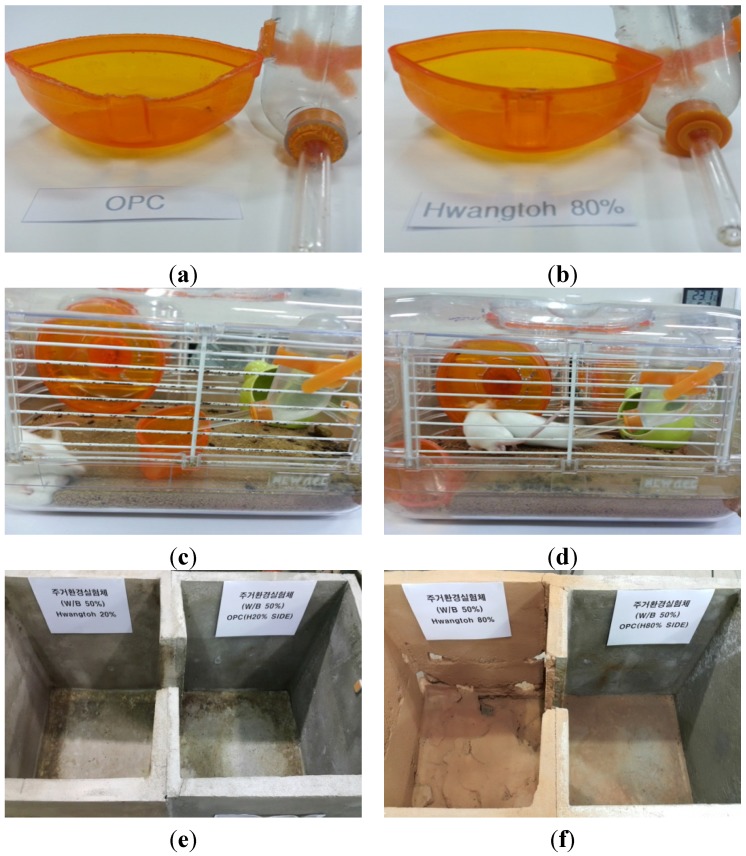
Damages caused by aggression. (**a**) Feeding and watering containers for OPC cage; (**b**) Feeding and watering containers for H80 cage; (**c**) Protection bars for OPC floor cages; (**d**) Protection bars for H20 floor cage; (**e**) Damaged lining of H20 residence cage, and (**f**) Damaged lining of H80 residence cage.

Funase (2002) reported that the level of stress and lifespan of subjects differs according to the type of construction material used in the subjects’ living space, such as timber, metal, or concrete. In particular, Funase suggested that concrete can reduce subjects’ lifespan by nine years as a result of the emissivity that occurs in the winter when the low external temperature is transmitted to the internal living environment through the walls [18]. Aggression is a particularly difficult behavior to quantitatively measure. In the present study, it was measured by examining the level of damage to the equipment and structures within the living spaces. The most aggressive behavior started after day 21 (the beginning of winter), and the emissivity was considered during the analysis. As shown in Figure 17, the structures within the OPC cast were more damaged than those within H80. In the residential environment selection experiment, the structures in the H20 cast were not damaged at all; however, the structures in the H80 cast sustained significant damage. Thus, as the Hwangtoh admixture ratio increased, the ICR mice felt the H80 floors and walls to be similar to soil, which brought out their natural digging and scratching instincts.

### Fertility Rate

**Figure 18:**
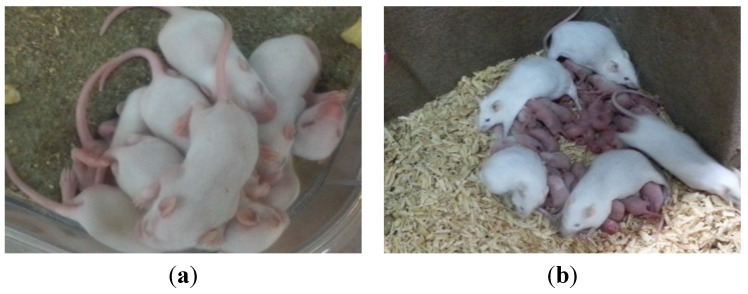
Breeding of ICR. (**a**) Floor cages and (**b**) Residence cage.

**Figure 19:**
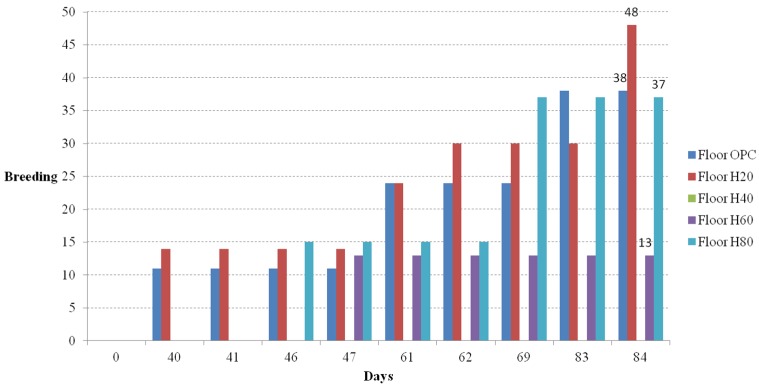
Breeding *versus* time curve (floor cages).

**Figure 20:**
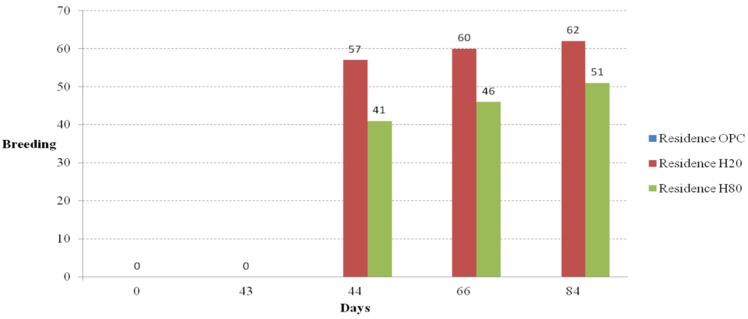
Breeding results curve (residence cages).

The ICR mice used in this experiment were fully fertile three weeks after birth, and indeed the females were pregnant around day 21 after birth. The fertility rates of the ICR mice in the five casts with different floors and two casts with different living environments were compared. Photos of the offspring from the floor cage and residence selection casts are shown in Figure 18a,b, respectively. The number of births for the floor cages and the residence selection casts are shown in Figure 19 and Figure 20, respectively, as bar graphs. The regular and frequent pregnancy and reproduction shows that the amount of food and water provided was ample.

## 6. Residential Environment Selection

**Figure 21:**
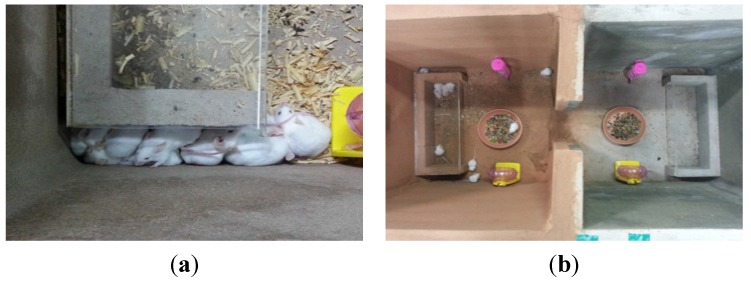
Residence preference. (**a**) 20% Hwangtoh residence cage and (**b**) 80% Hwangtoh residence cage.

ICR mice were allowed to choose between two different living environments, one with an activated Hwangtoh mixed mortar and the other with an ordinary cement mortar to examine their choices over 12 weeks. Equal amounts of food and water were provided in each environment. As shown in Figure 21, in the first experiment, all of the mice chose the activated Hwangtoh environment within three hours of testing. They maintained their selection in repetitive experiments. Furthermore, although it initially took three hours for the mice to move to their chosen environment, the selection time grew shorter with each follow-up experiment. In the last experiment, it took less than one minute for some of the mice to move to their chosen environment. At the end of the experiment, the amount of leftover food and water in the activated Hwangtoh environment was considerably less than that in the OPC environment. Under an equal external temperature, the temperature and humidity within the activated Hwangtoh cast and OPC cast differed by 0.1 °C–1.5 °C and 1.0%–5.0%, respectively. To determine whether the differential amount of food and water intake in the two environments was caused by the differences in temperature and humidity or by hazardous components in the cement, the temperature and humidity in the OPC cast were varied to match those in the activated Hwangtoh environment. Even under identical temperature and humidity levels, the ICR mice maintained their choice of environment. This behavior indirectly indicates that the lower amount of food and water intake in the normal mortar environment was caused by harmful components in the cement rather than the difference in relative temperature and humidity. In a further study, in-depth research should be performed to examine the cement components that could pose risks to human health.

## 7. Conclusions

The following conclusions are drawn from this study.

(1) Although the activated Hwangtoh mixed concrete showed slightly lower compressive strength than the ordinary concrete, it is a suitable construction material, except in those cases that require exceptionally high strength and durability.
(2) Because the activated Hwangtoh mixed concrete tended to absorb more water, the optimal addition rate of superplasticizer for better workability was 1.5 volume %.
(3) The pH of the activated Hwangtoh mixed concrete was lower than that of ordinary concrete.
(4) The differences in temperature and relative humidity between OPC mortar and Hwangtoh cement mortar measured from the residence selection casts were 0.1 °C–1.5 °C and 1.0%–5.0%, respectively.
(5) The amount of food and water intake by the mice in the activated Hwangtoh environment was considerably higher than that in the OPC environment. Also, the mice in the activated Hwangtoh environment had a higher fertility rate and healthier offspring.
(6) The ICR mice moved to the ordinary cement cast only for food and water. At all other times, they stayed in the activated Hwangtoh living quarter.
